# A pipeline to evaluate inhibitors of the *Pseudomonas aeruginosa* exotoxin U

**DOI:** 10.1042/BCJ20200780

**Published:** 2021-02-12

**Authors:** Daniel M. Foulkes, Keri McLean, Yalin Zheng, Joscelyn Sarsby, Atikah S. Haneef, David G. Fernig, Craig Winstanley, Neil Berry, Stephen B. Kaye

**Affiliations:** 1 Department of Eye and Vision Science, Institute of Life Course and Medical Sciences, University of Liverpool, Liverpool, U.K.; 2 Department of Biochemistry, Institute of Integrative Biology, University of Liverpool, Liverpool, U.K.; 3 Department of Clinical Infection, Microbiology and Immunology, University of Liverpool, Liverpool, U.K.; 4 Department of Chemistry, University of Liverpool, Liverpool, U.K.

**Keywords:** antimicrobial, ExoU, inhibitor, *Pseudomonas aeruginosa*, type 3 secretion system, virulence factor

## Abstract

*Pseudomonas aeruginosa* has recently been highlighted by the World Health Organisation (WHO) as a major threat with high priority for the development of new therapies. In severe *P. aeruginosa* infections, the phospholipase activity of the type 3 secretion system toxin, ExoU, induces lysis of target host cells and results in the poorest clinical outcomes. We have developed an integrated pipeline to evaluate small molecule inhibitors of ExoU *in vitro* and in cultured cell models, including a disease-relevant corneal epithelial (HCE-T) scratch and infection model using florescence microscopy and cell viability assays. Compounds Pseudolipasin A, compound A and compound B were effective *in vitro* inhibitors of ExoU and mitigated *P. aeruginosa* ExoU-dependent cytotoxicity after infection of HCE-T cells at concentrations as low as 0.5 µM. Addition of the antimicrobial moxifloxacin controlled bacterial load, allowing these assays to be extended from 6 h to 24 h. *P. aeruginosa* remained cytotoxic to HCE-T cells with moxifloxacin, present at the minimal inhibitory concentration for 24 h, but, when used in combination with either Pseudolipasin A, compound A or compound B, a greater amount of viable cells and scratch healing were observed. Thus, our pipeline provides evidence that ExoU inhibitors could be used in combination with certain antimicrobials as a novel means to treat infections due to ExoU producing *P. aeruginosa*, as well as the means to identify more potent ExoU inhibitors for future therapeutics.

## Introduction

*Pseudomonas aeruginosa* is a motile, Gram-negative bacterium that causes a wide range of opportunist infections including ocular, soft tissue, urinary tract and respiratory tract infections [1–5]. It is a major cause of intensive care unit-acquired pneumonia (ICUAP), as well as a known coloniser of patients with cystic fibrosis and those who are immunocompromised [6]. ICUAP could also contribute to mortality in patients with COVID-19 and patients are currently being recruited for a clinical trial to test this hypothesis [7]. In the eye, *P. aeruginosa* accounts for ∼25% of cases of bacterial keratitis and it is the most common causative agent of bacterial keratitis associated with contact lens use. After cataracts, bacterial keratitis is the second largest cause of legal blindness worldwide [8]. Moreover, multidrug-resistant *P. aeruginosa* is a major threat, as recently highlighted when the World Health Organisation (WHO) listed carbapenem-resistant *P. aeruginosa* with the highest priority for the development of new antibiotics [9]. It is, therefore, imperative that efforts are made to develop novel treatments to target this pathogen.

During infection, the pathogen exploits its large genome, encoding complex regulatory networks and a wide range of virulence factors, including exotoxins. *P. aeruginosa* employs a needle-like apparatus, called the type 3 secretion system (T3SS) that extends through the cell wall to the outer membrane, which it uses to inject certain exotoxins directly into the target cell cytosol [10,11]. Of the four effector proteins, ExoS, ExoT, ExoU and ExoY, the most cytotoxic is ExoU. In *P. aeruginosa* infections where the ExoU gene is expressed, disease severity is increased with poorer clinical outcomes [4,5,12,13]. This is considered to be due to rapid cell lysis, mediated by the phospholipase activity of ExoU, which targets the host cell plasma membrane from the cytosol and cannot be halted before conventional antimicrobials can successfully eliminate the pathogen [14,15].

ExoU is a 74-kDa soluble protein that possesses an N-terminal bacterial chaperone interacting domain followed by a patatin-like phospholipase (PLP) domain and finally a C-terminus containing a 4-helical bundle, which is employed for ExoU insertion into plasma membranes [16–18]. The two currently reported crystal structures of ExoU (3TU3 [19] and 4AKX [20]) consist of ExoU bound to its cognate chaperone, SpcU, in a catalytically inactive complex. To date, it has not been possible to crystallise ExoU alone in the absence of its chaperone. This may be due to the fact that ExoU alone is too flexible to crystallise; protein flexibility being a structural requirement for passage through the Type III secretion system. Site-directed spin labelling (SDSL) [21] and double electron–electron resonance (DEER) studies [19] suggest that ExoU adopts multiple conformational states in the absence of activating cofactors, which may explain why ExoU has not yet been crystallised alone.

Although the mechanisms of ExoU activation have yet to be fully explored, it is established that certain eukaryotic host cofactors directly interact with ExoU and are required for the induction of its catalytic phospholipase activity [15]. Thus, ExoU relies on non-covalent binding to ubiquitin and phosphatidylinositol 4,5-bisphosphate (PIP_2_) to become fully activated [22,23]. Between the phospholipase domain and C-terminal 4-helical bundle of ExoU, there is a so-called bridging domain (amino acids 480–580), which forms the proposed binding site for ubiquitin [23,24]. ExoU phospholipase activity can be detected *in vitro* in the presence of ubiquitin as an activating cofactor [25]. In the presence of PIP_2_, ExoU forms multimers and its ubiquitin-dependent catalytic activity is greatly enhanced [26]. In mammalian cells, binding of PIP_2_ also serves to localise ExoU to the plasma membrane, via the 4-helical bundle domain, where it oligomerises and its catalytic activity induces cellular lysis [22,27,28].

The multiple dynamic conformational changes that occur during ExoU activation may be targeted by small molecules to attenuate ExoU activity in clinical infections [15]. Such a strategy would reduce tissue damage and inflammation associated with infection and provide a more substantial window for immune response or antibiotic action. Previous work has used cell-based assays to screen for small molecule inhibitors of ExoU. An integrated pipeline from an *in vitro* enzyme screen using pure recombinant ExoU through cell models of decreasing throughput, but increasing clinical relevance, coupled to molecule docking of inhibitors, however, is lacking. Here we develop such a pipeline which necessitated overcoming some of the challenges in the field, such as the production of pure recombinant active ExoU. This allowed the elaboration of an enzyme activity screen in multiwell plates. Alongside we used an inducible transfection model in HeLa cells, which identified inhibitors able to act on ExoU in the cytoplasm of a mammalian cell, and a disease-relevant co-infection scratch assay with human corneal epithelial (HCE-T) cells that reported on the ability of compounds to prevent ExoU mediated cell death and the degree of wound healing. This pipeline demonstrated that a proposed ExoU inhibitor identified in a high-throughput yeast assay [29], arylsulfonamide 1, neither inhibited ExoU *in vitro* nor protected transfected HeLa cells or HCE-T cells from ExoU cytoxicity. In addition, we were able to rank compounds identified by other means in terms of their efficacy *in vitro* and in the cell models, as well as predict their likely molecular contacts with ExoU.

## Materials and methods

### Chemicals, reagents and antibodies

Tetracycline (TET), MG132, PMSF, tobramycin and moxifloxacin, α-FLAG and α-tubulin antibodies, Pseudolipasin A (PSA), 5,5′-dithio-bis-(2-nitrobenzoic acid) (DNTB) and bovine ubiquitin were purchased from Sigma–Aldrich. The pOPIN bacterial expression vectors were purchased from Addgene. Compounds A (2-[(3-chlorophenyl)amino]-4,6-dimethylnicotinamide) and B 2-[(2,5-dichlorophenyl)amino]-4,6-dimethylnicotinamide were purchased from ChemBridge. Arylsulfonamide 1 was purchased from MolPort. Quinacrine dihydrochloride (QD) and oleyoxylethyl phosphorylcholine (OP) were purchased from Santa Cruz Biotechnology. Phosphatidylinositol 4 5-bisphosphate (PIP_2_) was purchased from Avanti polar lipids.

### *Pseudomonas aeruginosa* strains and mutants used in this study

The *P. aeruginosa* strains and mutants used in this study have been described previously [16,30,31], and were a kind gift from Professor Dara Frank (Medical College of Wisconsin). The ExoU producing strain of *P. aeruginosa*, PA103, and an effector null mutant, which lacks both ExoU and ExoT (PA103ΔUT) were used as positive and negative controls. The PA103ΔUT mutant, when complemented with a pUCP18 plasmid containing the ExoU gene (PA103ΔUT: WT ExoU), fully restores cytotoxic activity towards eukaryotic cells. When the PA103ΔUT mutant is transformed with pUCP18 plasmid encoding the catalytically inactive S142A ExoU variant (PA103ΔUT: S142A ExoU), acute cytotoxicity is not observed after infection of mammalian cells. *P. aeruginosa* transformed with pUCP plasmids were grown on agar or in LB broth supplemented with 300 μg/ml carbenicillin.

### Recombinant protein production

Full-length ExoU was cloned into pOPINF to generate a His-ExoU encoding construct. Expression conditions were adapted and optimised from previously established protocols [26,32,33]. C43(DE3) bacteria were grown in 1 l of Terrific broth (Melford) supplemented with ampicillin (100 μg/ml) and grown to an optical density (OD_600_) of 0.8 at 30°C before induction of ExoU expression with 0.4 mM isopropyl-β-d-thiogalactopyranoside (IPTG). Phenylmethylsulfonyl fluoride (PMSF) (100 µM) (0.1% v/v ethanol) was also added to *Escherichia coli* each hour over 3 h of induced ExoU expression. The bacterial lysis buffer contained 20 mM Tris–HCl pH 8.2, 300 mM NaCl, 0.1% (v/v) Triton-X-100, 10 mM imidazole, 1 mM DTT, 10% (v/v) glycerol and a cOmplete protease inhibitor cocktail tablet (Roche) and 100 µM PMSF. ExoU was purified by an initial affinity step (immobilised nickel affinity chromatography) followed by size-exclusion chromatography (SEC) (16/600 Superdex 200, GE healthcare) in 20 mM Tris–HCl pH 8.2, 100 mM NaCl and 10% (v/v) glycerol. Recombinant ExoU was frozen in liquid nitrogen and stored at −80°C.

### Mass spectrometry

After proteins were resolved by SDS–PAGE and stained with Comassie, gel bands were excised and de-stained using alternating solutions of 25 mM NH_4_HCO_3_ in 2 : 1 water/ACN and 25 mM NH_4_HCO_3_ incubated at 37°C for 15 min each. Gel pieces were incubated in 10 mM DTT for 60 min at 60°C, the solution discarded and then gel pieces were incubated in 55 mM iodoacetamide for 45 min at room temperature in the dark. After the iodoacetamide was discarded, gel pieces were washed twice in 25 mM NH_4_HCO_3_, dehydrated by washing in acetonitrile and left to air dry. Gel pieces were cooled on ice and rehydrated with 100 µl of 10 ng/µl Trypsin Gold (Promega, U.K.) for 5 min and then incubated overnight at 37°C. Digestion was terminated by adding formic acid to a final concentration of 1% (v/v). The solution was removed and retained. Gel pieces were then incubated in 10% (v/v) formic acid for 45 min and this solution was combined with the previous one. The final extract was dried in a vacuum centrifuge until nearly dry and re-suspended in 20 µl of 97 : 3 water : acetonitrile with 0.1% (v/v) TFA.

Peptides were analysed on an Ultimate 3000 RSLCTM nano-LC (Thermo Scientific, Hemel Hemstead) coupled to a QExactiveTM mass spectrometer (Thermo Scientific). Peptides from bands 1, 2 and 5 were diluted 10- fold in 97:3 water : ACN + 0.1% (v/v) TFA and 1 µl of this dilution and 1 µl of the undiluted peptides from bands 3 and 4 were injected onto the trapping column (Thermo Scientific, PepMap100, C18, 300 µm × 5 mm), using a partial loop injection, for 7 min at a flow rate of 4 µl/min with 0.1% (v/v) TFA and then resolved on an analytical column (Easy-Spray C18, 75 µm × 500 mm, 2 µm bead diameter) using a gradient of 97% A (0.1% v/v) formic acid in H_2_O) and 3% B (0.1% (v/v) formic acid in 80 : 20 ACN:H_2_O) to 50% B over 15 min at a flow rate of 300 nl/min. A full-scan mass spectrum was acquired over 350–2200 m/z, AGC set to 3e6, with a maximum injection time of 100 ms. The top 3 peaks were selected for MS/MS with an ion selection window of 1.2 m/z and a normalised collision energy of 30, the AGC was set to 1e4 and a maximum injection time of 45 ms. To avoid repeat selection of peptides a 20 s exclusion window was used.

Data were processed using Proteome Discover 1.4 (Thermo Scientific) and searched using Mascot against an E.coli protein database (retrieved from Uniprot-reviewed proteome accessed May 2015), a database of known contaminates (including common proteases and keratins) and the ExoU protein.

### *In vitro* PLA_2_ assay

The protocol from the Cayman Chemical (U.S.A., Michigan) cPLA_2_ assay kit was adapted, which allowed the analysis of ExoU SN2 directed phospholipase activity in the presence of compounds in both 96 and 384-well plate formats. Substrate arachidonoyl thio-phosphatidylcholine (ATPC) was purchased from Cayman Chemical as an ethanolic solution. The ethanol was evaporated under a gentle stream of nitrogen gas prior to the dissolution of ATPC in 80 mM Hepes pH 7.4, 150 mM NaCl, 4 mM Triton-X-100, 30% (v/v) glycerol and 1 mg/ml bovine serum albumin (BSA) to yield a 1.5 mM substrate stock solution. The final reaction mixture contained 1 µM PIP_2_, 25 µM ubiquitin, 1 mM ATPC substrate, 2% (v/v) dimethylsulfoxide (DMSO) (with or without compound) and 1.25 mM 5,5-dithio-bis-(2-nitrobenzoic acid) (DTNB) (dissolved in Mili-Q water) to which 1 µM of ExoU was added to initiate substrate hydrolysis. The absorbance at 405 nm (A405) was measured with the subtraction of the background absorbance (substrate and DTNB alone) at 10 minute increments over 12 h. Substrate hydrolysis was calculated using the equation A405/10.00 × 0.05 ml/number of nanomoles of ExoU for the 96-well plate format and A405/10.00 × 0.01 ml/number of nanomoles of ExoU for 384-well plate format, where 10.00 was the path length-adjusted extinction coefficient of DTNB and 0.05 (96-well plate) and 0.01 (384-well plate) were the reaction volumes in millilitres.

### Hela transfections

Adherent parental Flp-In T-REx-HeLa (Invitrogen) were cultured in Dulbecco's modified Eagle medium (DMEM) supplemented with 4 mM l-glutamine, 10% (v/v) foetal bovine serum (FBS), Penicillin and Streptomycin (Gibco), 4 μg/ml of Blasticidin (Melford) and Zeocin 50 μg/ml (Invitrogen). HeLa cells (2.2 × 10^6^ cells in 10 cm dishes and 0.5 × 10^6^ cells for 6-well plates) were seeded 24 h prior to transfection. For transient transfections, pcDNA5/FRT/TO plasmid encoding Flag-tagged WT ExoU or S142A ExoU was incubated in serum-free medium containing lipofectamine 2000 (Invitrogen) for 30 min at ambient temperature. The DNA lipofectamine mixture was then added to 10 cm dishes (for Western blot) or 6-well plates (for LDH, microscopy, trypan blue and propidium iodide uptake) of HeLa cells for 12 h. The cells were then washed with phosphate-buffered saline (PBS) and fresh medium containing 1 µg/ml TET (to induce ExoU expression) and DMSO (0.1% v/v) or the indicated compound was added for the indicated times.

### LDH assays

Lactate dehydrogenase (LDH) release was measured using the Pierce LDH Cytotoxicity Assay Kit (Thermo Scientific) according to the manufacturer's instructions. For HeLa cell experiments, culture medium (50 µl) was assayed at the indicated time points after transfection and induction of ExoU expression with TET, in the presence of specified compounds (0.1% v/v DMSO). The absorbance of the negative controls (untransfected cells) was subtracted to yield the final absorbance values. Medium (50 µl) of HCE-T cells was assayed at various times after infection with the indicated strains of *P. aeruginosa* in the presence of compounds (0.1% DMSO, v/v). The results were reported as percent cell lysis normalised to a positive control (according to the manufacturer's instructions), which gave the maximum amount of observable cell lysis in an appropriate detectable range of absorbance.

### Trypan blue assay

Transfected HeLa cells were collected, after 8 h of TET induced ExoU expression, by combining the culture medium (to obtain suspended cells) and adherent cells released by trypsinisation. The resulting HeLa cell and medium suspension was mixed in a 1 : 1 ratio with trypan blue reagent (Thermo Scientific) and a Countess II Automated Cell Counter (Thermo Scientific) was employed to detect the percentage of cells with compromised membrane integrity.

### Propidium iodide uptake

Transfected HeLa cells (including suspended cells) were collected by trypsinisation 8 h after induction of ExoU expression in presence of the indicated compound. Samples were stained with propidium iodide (PI) and diluted with PBS so that samples contained less than 500 cells/µl for 10 000 events per run. After gating to select whole cells, employing a BD Accuri C6 flow cytometer, the total cell numbers were evaluated with forward scatter and PI-stained cells were detected by using an appropriate laser for fluorescence with results given as relative florescence units for PI uptake.

### Western blotting

HeLa whole-cell lysates were generated using a modified RIPA buffer (50 mM Tris–HCl pH 7.4, 1% (v/v) NP-40, 0.1% (v/v) SDS, 100 mM NaCl, 1 mM DTT, 10% (v/v) glycerol, a complete protease inhibitor cocktail tablet (Roche) and 100 µM PMSF. After resuspension in lysis buffer, HeLa cells were briefly sonicated and centrifuged at 16 000***g*** prior to quantification of protein concentration with the Bradford assay. Samples were boiled for 5 min in sample buffer (50 mM Tris–Cl pH 6.8, 1% (w/v) SDS, 10% (v/v) glycerol, 0.01% (w/v) bromophenol blue, and 10 mM DTT). Subsequently, 40 μg of total protein for each sample was resolved by SDS–PAGE prior to transfer to nitrocellulose membranes (Bio-Rad). Membranes were blocked in Tris-buffered saline with 0.1% (v/v) Tween 20 (TBS-T) in 5% (w/v) non-fat dried milk (pH 7.4) followed by incubation with primary and secondary antibodies. Proteins were detected using horse radish peroxidase-conjugated antibodies and enhanced chemiluminesence reagent (Bio-Rad). Band intensities were quantified using ImageJ software.

### HCE-T scratch and infection assay

HCE-T cells were seeded (0.5 × 10^6^) in 6-well plates and grown until a fully confluent monolayer had formed, at which point two parallel scratches were made across the diameter of the wells with a 10 µl pipette tip. In tandem, PA103 and PA103 mutant bacteria were inoculated into LB culture medium (with or without appropriate antibiotic selection marker) and grown overnight. Subsequently, 1 ml of the overnight bacterial culture was subcultured in 50 ml of fresh LB medium and allowed to expand until an OD_600_ of 0.8 was reached. At the point of compound addition to scratched HCT cells, indicated PA103 or PA103 mutant strains were added immediately at a multiplicity of infection (MOI) of 2.5. Where indicated, 2 µM of moxifloxacin was also added to control bacterial growth.

### MTT assays

HCE-T cells were seeded in a 96-well plate at a concentration of 0.2 × 10^6^ cells/ml and allowed to grow until a fully confluent monolayer had formed. Compound addition was performed in triplicate, with all experiments including a final concentration of 0.1% DMSO (v/v). Metabolic activity was quantified 48 h after compound addition, and cell viability was quantified employing an MTT assay kit (Abcam, place) according to the manufacturer's instructions. Briefly, thiazolyl blue tetrazolium bromide was dissolved in PBS and added to cells at a final concentration of 0.25 mg/ml and incubated at 37°C for 3 h. The reaction was stopped by the addition of 50 μl acidified 10% (w/v) SDS, followed by reading of absorbance at 570 nm. Viability was defined relative to DMSO-containing controls incubated for the same time.

### Fluorescence microscopy

HCE-T cells treated as indicated in the figure legends were incubated for either 8 h or 24 h before analysis by florescent microscopy, employing Live/Dead staining (Invitrogen), according to the manufacturer's instructions, in order to differentiate and visualise viable and dead/dying cells. Briefly, the culture medium was removed from the infected HCT cells in 6-well plates, which were then washed with 1 ml of PBS three times and fresh medium added, containing 5 µM of both calcein (Ex/Em 494/517 nm) and ethidium homodimer-1 (Ex/Em 528/617 nm). Images of the scratched HCT cells were obtained on either an Apotome Zeiss Axio Observer or a Nikon Eclipse TiE. Images were subject to automated analysis using an in-house program written in Matlab R2019a (Mathworks, Natick, US), described in [34,35]. The total number of dead/dying cells and viable cells were counted, as previously described [34]. For the quantification of the scratched area, each image was converted from colour to greyscale, and then segmented using a texture-based segmentation approach previously described [35]. After segmentation, the scratch area was calculated by multiplying the dimensions of each pixel not occupied by cells.

### Molecular docking simulations

The chemical structures of PSA, compound A and compound B were illustrated in ChemDraw and imported into Spartan18 where their 3D structures were energy minimised using the MMFF94 forcefield prior to docking experiments. The crystal structure of ExoU (bound to SpcU) (PDB: 3TU3) was imported into the molecular visualising software Hermes and the program GOLD 5.2 [36] was employed to model ligand docking to ExoU with the binding site of the protein defined as being within 10 Å of the catalytic Serine 142 residue of ExoU. Default settings were retained apart from ‘GA settings’ were changed to 200%. Protein and ligand docking poses were visualised in the PyMOL Molecular Graphics System, Version 2.0 Schrödinger. Non-covalent contacts were analysed with ViewContacts software [37].

### Statistical analysis

All experimental procedures were repeated in at least three separate experiments with matched positive and negative controls (unless stated otherwise) and results are presented as means ± SD. When applied, the statistical significance of differences (*P ≤ 0.05) was assessed using One-way ANOVA or Student's *t*-tests for normally distributed data. Statistical tests were performed using either SPSS or Prism 7 (GraphPad Software).

## Results

### Purification and analysis of recombinant exoU *in vitro*

**Figure 1.**
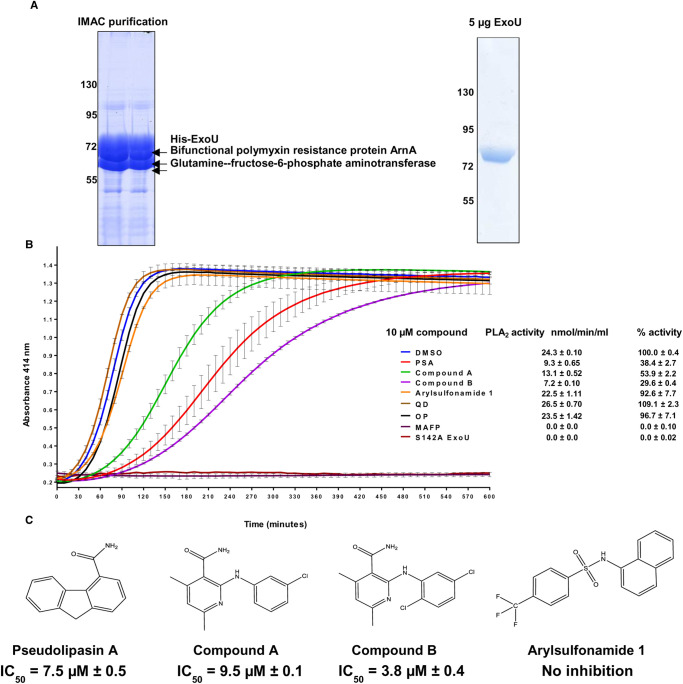
Purification and *in vitro* analysis of recombinant ExoU with prospective small molecule inhibitors. (**A**) Left: His-tagged ExoU was purified from C43(DE3) *E. coli*. Immobilised metal affinity chromatography (IMAC) purified His-ExoU, including two major contaminant proteins, were resolved by SDS–polyacrylamide gel electrophoresis (SDS–PAGE) and identified by employing mass spectrometry. Right: His-ExoU was further purified to homogeneity by size-exclusion chromatography; 5 µg of His-ExoU was resolved and visualised by SDS–PAGE. (**B**) The hydrolysis of arachidonoyl Thio-PC substrate by ExoU was assessed in the presence of 10 µM of the indicated compound. To each reaction, ubiquitin and PIP_2_ were added in order to allow induction of ExoU phospholipase activity. Experiments were performed in triplicate, the results represent means, and error bars represent standard deviations and representative profiles are shown of substrate conversion as a function of absorbance with the progression of time (QD = quinacrine dihydrochloride and OP = oleyoxylethyl phosphoryl choline). (**C**) Chemical structures of prospective compounds that are proposed to inhibit ExoU mediated toxicity in cells. IC_50_ values are shown.

To analyse the *in vitro* phospholipase activity of ExoU, we expressed ExoU with an N-terminal histidine tag in *E. coli* and purified it sequentially by immobilised metal affinity chromatography (IMAC) and then by SEC (Figure 1A). Building on previously optimised ExoU expression conditions [32,33], we added 100 µM of the serine protease inhibitor PMSF to *E. coli*, followed by the addition of IPTG to induce expression of ExoU for 3 h. This afforded enhanced yields of recombinant His-tagged ExoU (Supplementary Figure S1A). Without PMSF, typical yields per litre of *E. coli* culture were 0.1 mg. If, however, PMSF was added to IPTG induced *E. coli*, yields of 0.5 mg per litre could be achieved. SDS–PAGE showed that purification by IMAC yielded two major and one minor polypeptide of similar molecular size. Consequently, mass spectrometry was employed to elucidate their identity. This demonstrated that the minor band seen upon SDS–PAGE corresponded to ExoU, whereas the two major bands that migrated slightly faster were glutamine-fructose-6-phosphate aminotransferase (67 kDa) and bifunctional polymyxin resistance protein ArnA (74 kDa) (Figure 1A left). Interestingly, these two proteins were only present in *E. coli* after induction of ExoU expression, indicating that there were somehow linked to the latter (Figure 1A left). Importantly, despite similar molecular mass, these impurities could be separated from ExoU when it further purified by SEC (Figure 1A right).

Purified His-ExoU, analysed by SEC with reference to a series of molecular mass standards (Supplementary Figure S2), had a predicated molecular mass of ∼75 kDa, indicating that purified ExoU was monomeric. This was in accordance with a previous study which demonstrated, by SEC, that ExoU existed in a monomeric state unless induced to form higher-order molecular mass complexes by PIP_2_ [26]. We observed that a portion of ExoU eluted in the void volume (Supplementary Figure S2A), which was likely the result of partial protein aggregation.

The phospholipase activity of ExoU was measured by adaptation of the Cayman chemical PLA_2_ phospholipase assay kit, so that analysis was compatible with 96 and 384-well plate formats, in the presence of PIP_2_ and ubiquitin, cofactors necessary for ExoU activation. Arachidonoyl Thio-PC substrate hydrolysis by 1 µM of ExoU in 2% (v/v) DMSO was 24.3 ± 0.1 nmol/min/ml (Figure 1B). Serine 142 is indispensable for ExoU catalytic activity [16] and mutation to an alanine abolished detectable substrate hydrolysis (Figure 1B). The promiscuous, broad-spectrum PLA_2_ inhibitor MAFP, which covalently binds to the catalytic serine residue of target PLA_2_ enzymes, irreversibly inhibited ExoU [38,39] (Figure 1B). Previously identified ExoU inhibitors Pseudolipasin A (PSA), compound A and compound B [39] (Figure 1C), at a concentration of 10 µM, were inhibitors of ExoU catalytic activity *in vitro*, resulting in decreased rates of substrate hydrolysis to 20 ± 3.8, 40 ± 2.8 and 15 ± 1.1 nmol/min/ml, respectively (Figure 1B). Dose–response analysis was performed for compounds PSA, compound A and compound B in order to establish IC_50_ values for inhibition, which were 7.5 ± 0.5, 9.5 ± 0.1 and 3.8 ± 0.4 µM, respectively.

In contrast, arylsulfonamide 1, which was previously identified from a high-throughput screen to protect yeast after infection with *P. aeruginosa* (with ExoU as only virulence effector) [29] did not inhibit recombinant ExoU phospholipase activity *in vitro* (Figure 1C). Thus, the observed protection of yeast cells by arylsulfonamide may not have been due to a direct effect of the compound on ExoU. In addition, we tested two non-specific inhibitors of human PLA_2_ enzymes, QD and OP, which also did not inhibit *in vitro* ExoU activity

### Targeting of ExoU with prospective inhibitors in a HeLa cell model

Flp-In T-REx-HeLa cells were transfected with pcDNA5FRT/TO, encoding either WT or S142A ExoU cDNA, so that FLAG-tagged-ExoU could be expressed upon addition of TET. Transfected HeLa cells induced to express WT ExoU underwent rapid cellular lysis within 8 h, whereas cells expressing S142A ExoU remained intact (Supplementary Figure S3A). This meant that only S142A ExoU could be reasonably observed by immunoblotting analysis (exploiting the N-terminal flag tag) of the cell extracts (Figure 3A) [40]. If transfected HeLa cells were TET induced and exposed to DMSO or the proteasome inhibitor MG132 for 4 h, FLAG-WT ExoU could not be detected (Supplementary Figure S4). FLAG-tagged WT-ExoU could, however, be detected if TET induced HeLa cells were incubated with 50 µM of the protease inhibitor PMSF for 4 h (Supplementary Figure S4).

**Figure 2.**
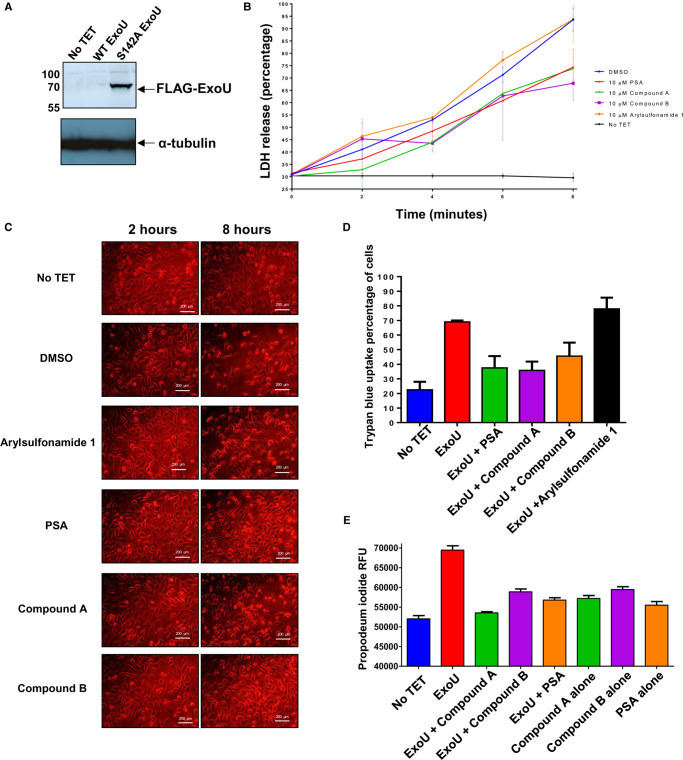
Inhibition of ExoU in a HeLa cell transfection model with prospective small molecules. (**A**) Flp-In T-REx-HeLa cells were transfected with pcDNA5/FRT/TO encoding the full-length WT or S142A EoxU gene so that FLAG-ExoU could be expressed upon incubation with TET for 6 h, prior to whole-cell lysis and analysis by western blotting. (**B**) LDH release of HeLa cells transfected and induced to express WT ExoU over 8 h in the presence of 10 µM of the indicated compound. One-way ANOVA analyses were performed to determine statistical significance between DMSO and compound treated cells. (**C**) Brightfield microscopy images of HeLa cells 8 h subsequent to induction of WT ExoU expression in the presence of indicated compound. (**D**) Trypan blue uptake of HeLa cells 8 h subsequent to induction WT ExoU expression in the presence of the indicated compound. (**E**) Propidium iodide uptake of WT ExoU expressing HeLa cells in the presence of the indicated compound, measured by flow cytometry.

To assess the efficacy of prospective ExoU inhibitors, transfected HeLa cells were induced to express WT ExoU by the addition of TET for 8 h in the presence of 10 µM of either PSA, compound A, compound B or arylsulfonamide 1; LDH release was assayed to quantify cell lysis (Figure 2B). With no inhibitor treatment (DMSO-blue) a steady increase in LDH release, up to the maximum detectable activity, was observed over the 8 h time course. The compound arylsulfonamide 1 (orange) afforded no protection from WT-ExoU mediated cell lysis, whereas PSA (red), compound A (green) and compound B (purple), significantly decreased the quantity of LDH activity detected in the culture medium over 8 h (*P*-values of 0.01, 0.01 and 0.02). Brightfield microscopy images (Figure 2C) complemented these findings by revealing observable morphological changes to the transfected HeLa cells when WT-ExoU was expressed. This included cell rounding and membrane blebbing (Figure 2C) [31]. Expression of WT-ExoU, without inhibitor treatment (DMSO), also correlated with fewer cells adhered to the well. Similar cellular morphologies were observed for HeLa cells induced to express ExoU in the presence of 10 µM arylsulfonamide 1, indicating that this compound had no substantial effect on ExoU inhibition, consistent with its lack of effect on ExoU activity *in vitro* (Figure 1C). Treatment with either 10 µM PSA, compound A or compound B resulted in more adherent cells with

Trypan blue and PI cellular uptake were employed to further assess cell lysis in transfected HeLa cells, induced to express ExoU after 8 h in the presence of prospective inhibitors. Consistently, the percentage of cells that absorbed the trypan blue dye was increased when HeLa cells were induced to express ExoU (Figure 2D). Compounds PSA, A and B, but not arylsulfonamide 1 caused a reduction in the uptake of trypan blue in induced HeLa cells. From a baseline uptake of PI, when ExoU expression was not induced (no TET), there was a significant increase PI florescence in cells induced to express ExoU (TET) (Figure 2E). This uptake was abrogated in the presence of either 10 µM PSA, compound A or compound B, but not arylsulfonamide 1.

**Figure 3.**
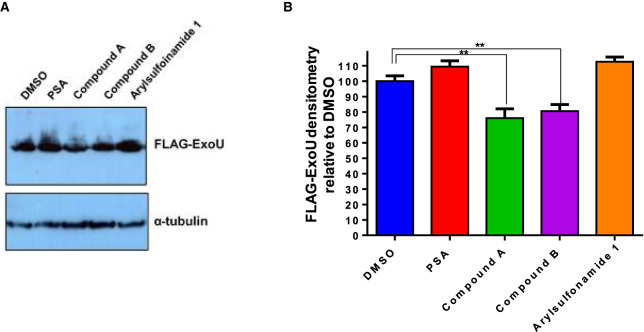
Analysis of S142A ExoU stability in HeLa cells in the presence of compound. (**A**) Flp-In T-REx-HeLa cells were transfected with pcDNA5/FRT/TO encoding the full-length S142A EoxU gene FLAG-S142A ExoU expression was induced with TET and 10 µM of the indicated compound for 8 h, prior to whole-cell lysis and analysis by Western blotting. (**B**) Densitometry analysis of S142A ExoU signal from compound treated HeLa cells, relative to a DMSO. *T*-tests were used to determine statistically significant differences between compound A (*P *= 0.0035) and compound B (*P *= 0.0039) relative to DMSO treated.

Western blot analysis was employed to determine the potential effects of compounds on ExoU stability in HeLa cells. As WT-ExoU was not readily detectable in whole-cell extracts (Figure 2A), we expressed FLAG-tagged S142A ExoU, which was detected in cellular lysates by immunoblotting, after induction of expression by TET in HeLa cells and incubation with chosen compounds for 8 h. After transfection with pcDNA5/FRT/TO, encoding FLAG-tagged S142A ExoU, and induction with TET for 8 h in the presence of compound, we observed a decrease in the level of S142A ExoU only in the presence of compounds A and B (Figure 3A). PSA, arylsulfonamide 1 and MG132 did not affect the quantity of S142A ExoU present in cell lysates in comparison with DMSO. The difference in total S142A ExoU protein, when quantified by densitometry, was significantly less for compound A (*P* = 0.005) and compound B (*P* = 0.008) compared with DMSO (0.1% v/v) alone.

### Establishment of a scratch assay to assess toxicity of ExoU expressing *Pseudomonas aeruginosa*

**Figure 4.**
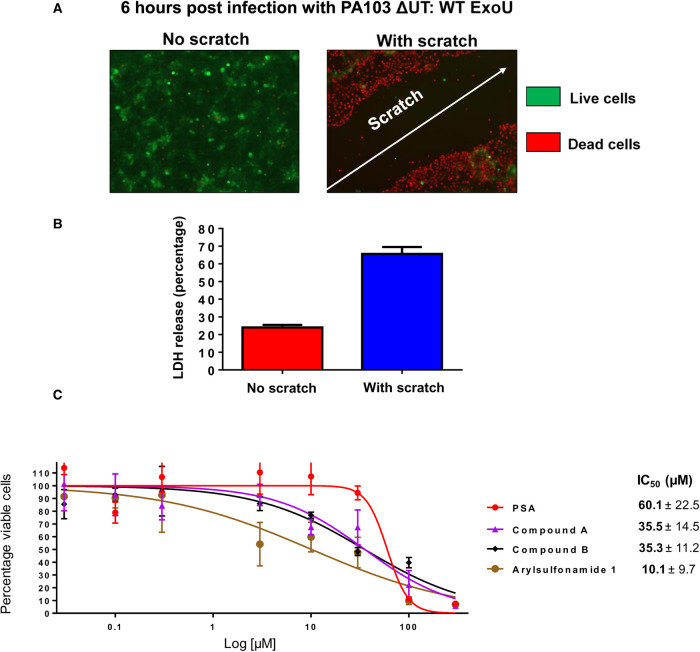
Establishment of a HCE-T cell scratch and infection assay with cytotoxicity analysis of prospective ExoU inhibitors. HCE-T were gown to full confluence and a scratch applied to the cell monolayer prior to infection with PA103 ΔUT: ExoU at an MOI of 2.5 for 6 h, followed by analysis by Live/Dead fluorescence microscopy (**A**) or LDH release (**B**). (**C**) MTT assays comparing the cytotoxicity of ExoU inhibitors in HCE-T cells. The MTT assay was performed 72 h subsequent to initial compound exposure and IC_50_ values in µM ± SD derived from three independent experiments are shown.

Expanding on established infection models of eukaryotic cells in culture with ExoU expressing *P. aeruginosa* [30,31], we developed a human corneal cell (HCE-T) scratch and infection assay. We employed previously utilised bacterial strains to assess the cytotoxic effects of ExoU in the HCE-T cells [30,31]. The ExoU expressing clinical isolate strain of *P. aeruginosa*, PA103, an ExoU and ExoT knock-out mutant (PA103 ΔUT) complemented with pUCP encoding either WT or S142A ExoU (PA103 ΔUT: ExoU and PA103 ΔUT: S142A ExoU), were used [30]. If either PA103 ΔUT: ExoU (which employs ExoU as the only T3SS cytotoxic effector) was added to fully confluent HCE-T cells, no effects of infection or ExoU mediated cytotoxicity could be detected after 6 h using fluorescence microscopy (Figure 4A) and measuring LDH activity (Figure 4B). However, if there was a defect in the monolayer or the monolayer was wounded by a scratch applied across the diameter of the well prior to infection, lysis of HCE-T cells induced by ExoU could be observed by florescence microscopy along the border of the scratch 6 h after initial infection with PA103 ΔUT: ExoU (Figure 4A). When the scratched HCE-T cells were infected with S142A ExoU expressing PA103 (PA103 ΔUT: S142A ExoU), there was a narrowing of the scratch (indicative of wound healing) and no observable dead cells adjacent to the scratch borders (Supplementary Figure S5A). This phenomenon was analogous to the control condition of no bacteria added to the scratched HCE-T cells and was reflected by a decrease in detectable LDH activity, relative to PA103 or PA103 ΔUT: WT ExoU infection (Supplementary Figure S5B).

MTT assays were used to assess the potential cytotoxicity of prospective ExoU small molecule inhibitors, which revealed that PSA, compound A and compound B were tolerated at the experimental concentrations used, with IC_50_ values of 60.1 ± 22.5, 35.5 ± 14.5 and 35.3 ± 11.2 µM (Figure 4C). The only compound that induced toxicity at assay working concentrations was arylsulfonamide 1 (brown), which exhibited an IC_50_ value of 10.1 ± 9.7 µM in the MTT assays (Figure 4B).

### Exou inhibitors mitigate cell lysis in a HCT-E scratch and infection assay

**Figure 5.**
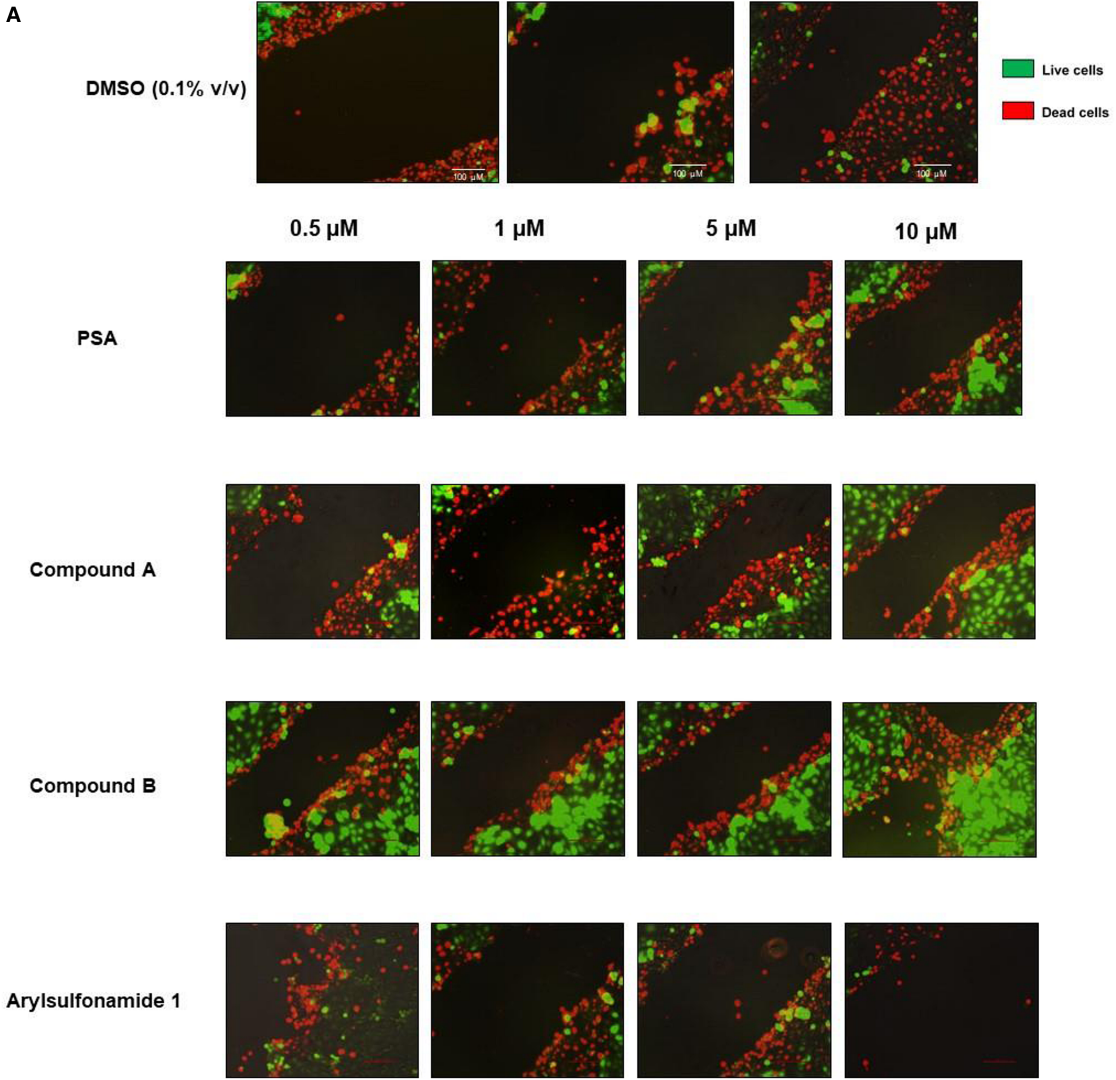

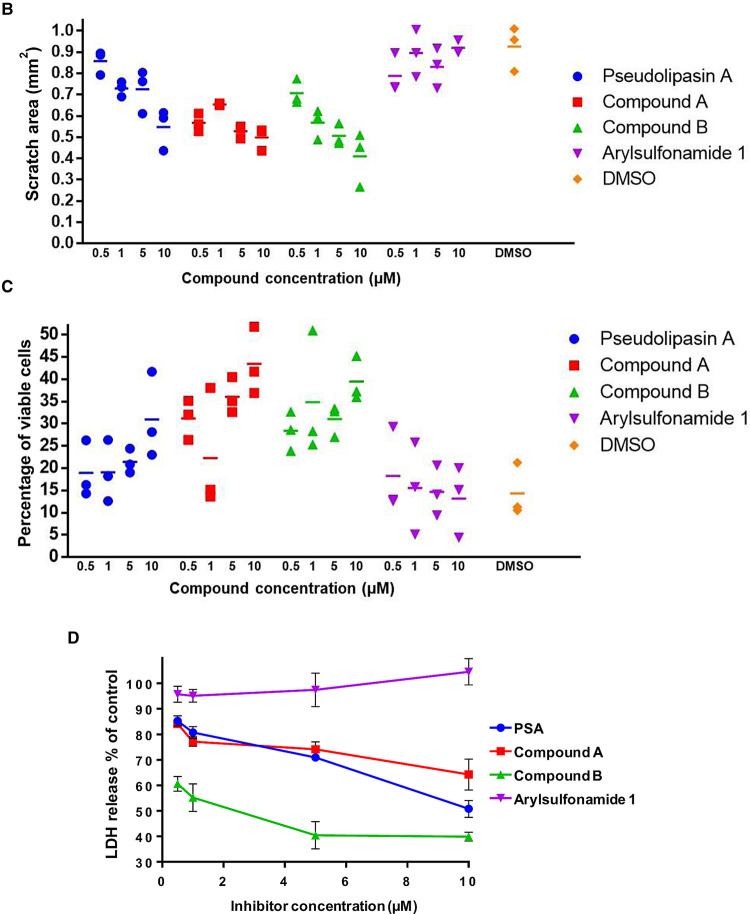
Protection of scratched HCE-T cells, during infection with ExoU expressing PA103, by selected compounds. (**A**) Live/Dead fluorescence microscopy analysis of scratched HCE-T cells 6 h post infection with ExoU expressing PA103 (PA103 ΔUT ExoU), in the presence of varying concentrations of indicated compound. (**B**) Measurement of total scratch area (mm^2^) in compound treated HCE-T cells 6 h post infection. (**C**) Percentage of viable cells calculated within the scratch margin. (**D**) Dose response analysis of inhibitors analysing the protective effect of compounds on scratched then infected HCE-T cells after 6 h incubation, by LDH release.

The scratch and infection assay was subsequently used to assess the ability of prospective small molecules to mitigate cytotoxicity induced by ExoU. Live/Dead fluorescence microscopy was performed on scratched HCE-T cells, 6 h after infection with PA103 ΔUT: ExoU (Figure 5A). Decreases in wound size, as well as the presence of fewer dead cells along the border of the scratch, were observed for treatments of infected HCE-T cells with varying concentrations of PSA, compound A and compound B, compared with DMSO (Figure 5A). Consistent with a lack of inhibitory activity on recombinant ExoU phospholipase activity (Figure 1B) or inhibition of ExoU mediated cytotoxicity in HeLa cells (Figure 2B), arylsulfonamide 1 did not protect scratched HCE-T cells against ExoU expressing PA103 infection (Figure 5A). By measuring the area of the scratch after infection and compound treatments (Figure 5B), we found that there was a reduction in total scratch surface area in the case of PSA, compound A and compound B treatments, resulting smaller scratch areas as compound concentrations were increased (Figure 5B). HCE-T exposure to both compounds A and B, at 0.5 µM, resulted in significant reductions in wound size, compared with DMSO alone (*P* = 0.005 and 0.034). At 10 µM of PSA, compound A and compound B exposure, the wound sizes after infection were reduced by 41%, 46% and 56%, compared with DMSO (*P* = 0.010, 0.003 and 0.006).

The total percentage of viable cells within the scratch margin was also calculated (Figure 5C). For compounds A and B, at a concentration of 0.5 µM, a significantly greater proportion of viable cells was detected, 6 h after PA103 : ΔUT ExoU infection (*P* = 0.017 and 0.031). At 10 µM the percentage of viable cells within the scratch margin was 43% for compound A and 40% for compound B, which was significantly different from that seen with DMSO (14%) (*P* = 0.007 and 0.005).

LDH release, as an indicator of cell lysis, complemented our fluorescence microscopy analysis (Figure 5D). Pseudolipasin A, compound A and compound B, but not arylsulfonamide 1, all reduced LDH release from HCE-T cells 6 h after infection in a dose-dependent manner (Figure 5D). Compound B was associated with the greatest reduction in observed LDH release. At 0.5 µM, compound B afforded the protection of HCE-T cells from ExoU mediated cell lysis, apparent from a 40% reduction in LDH release, compared with no treatment (0.1% v/v DMSO control). Indeed, higher concentrations of compound resulted in enhanced protection of HCE-T cells; at 10 µM of PSA, compound A and compound B, there were 49%, 35% and 60% reductions in LDH release, compared with DMSO controls. Although there were significant reductions in total scratch area (Figure 5B) and LDH (Figure 5D) release for PSA treated cells, in this assay, the total number of viable cells within the scratch margin (Figure 5C) was not found to be significantly different from DMSO.

### Compounds combined with moxifloxacin reduce ExoU induced cytotoxicity over 24 h

**Figure 6.**
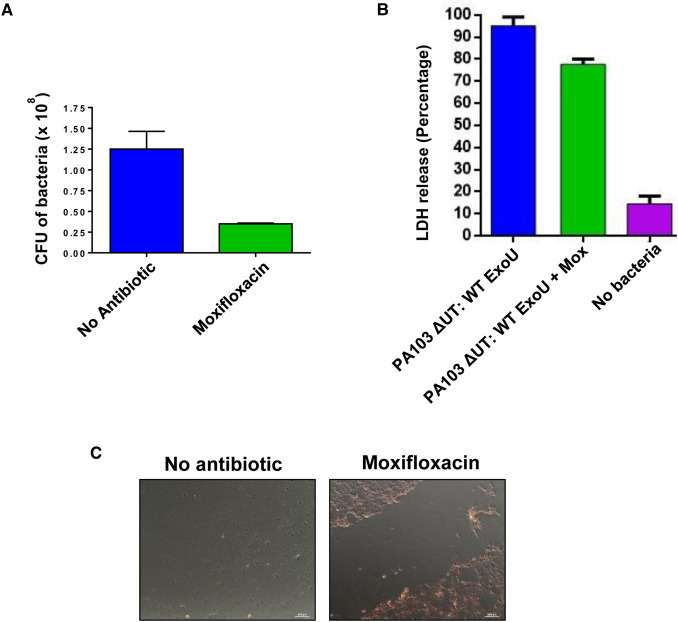

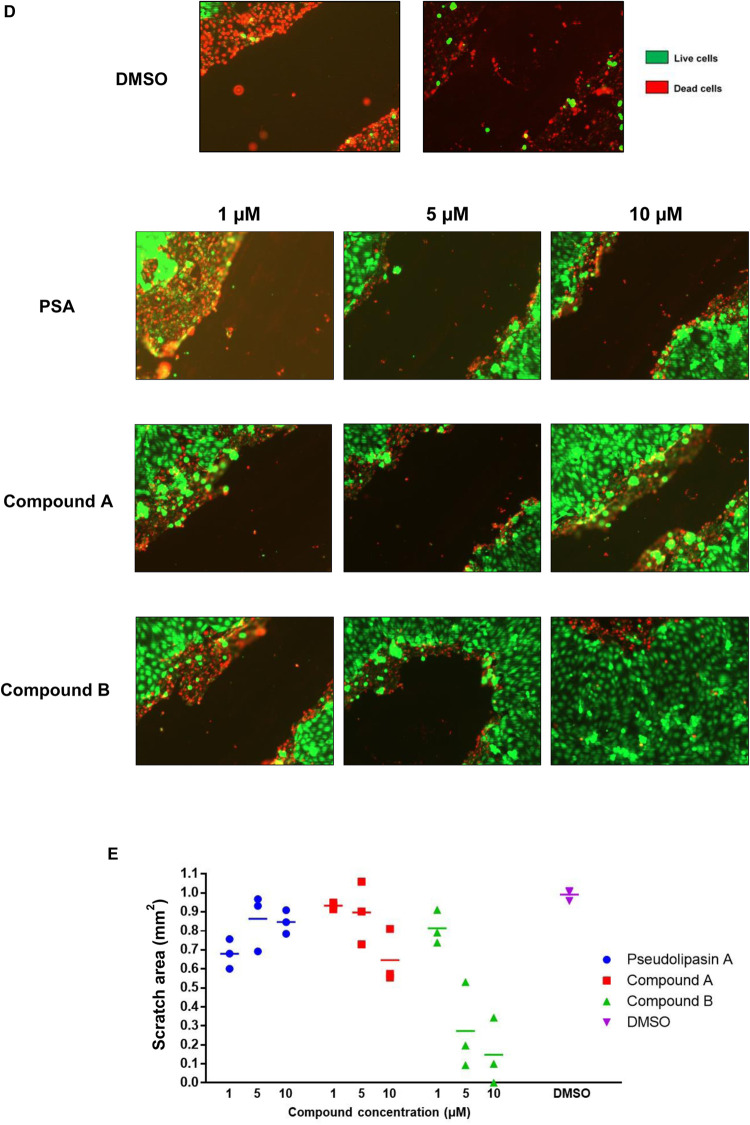

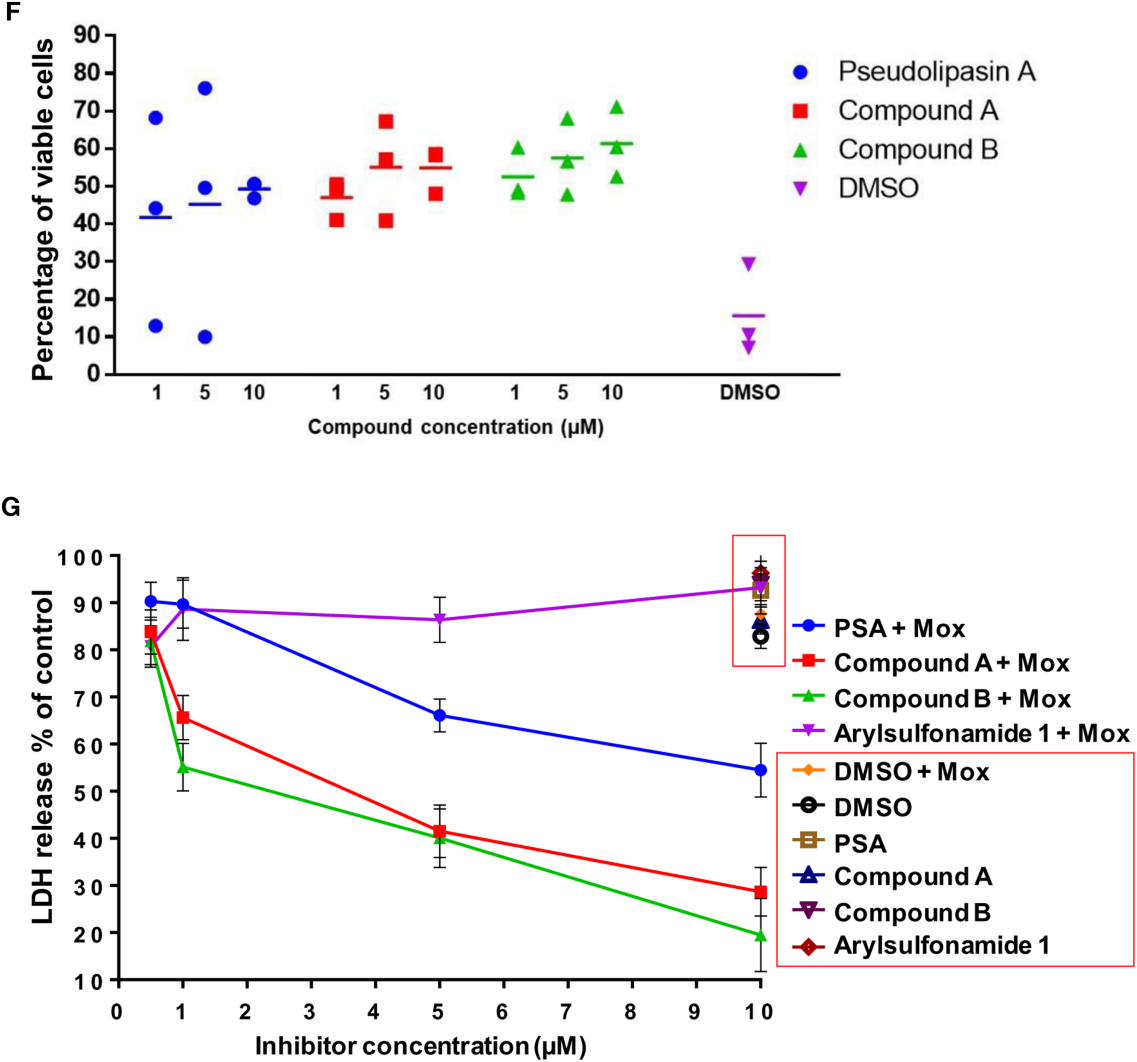
Compounds synergise with moxifloxacin to mitigate cell death induced by ExoU expressing PA103 over 24 h. (**A**) Moxifloxacin (Mox) at the established MIC of 2 µM was added to scratched HCE-T cells that had been infected with PA103 ΔUT: ExoU at an MOI of 2.5 for 24 h. The number of CFU in the cell culture medium was then deduced. (**B**) LDH release from scratched HCE-T cells after 24 h infection in the presence of moxifloxacin at the MIC. (**C**) Live/Dead fluorescence microscopy analysis of scratched HCE-T cells 24 h post infection, without and with moxifloxacin at the MIC. (**D**) Live/Dead fluorescence microscopy analysis of scratched HCE-T cells 24 h post infection, in the presence of varying concentrations of indicated compound, with moxifloxacin present at the MIC. (**E**) Measurement of total scratch area (mm^2^) in compound treated HCE-T cells 24 h post infection in the presence of moxifloxacin. (**F**) Percentage of viable cells calculated within the scratch margin 24 h after infection in the presence of moxifloxacin. (**G**) LDH assay for dose response analysis of inhibitors analysing protective effect of compounds on scratched then infected HCE-T cells after 24 h incubation in the presence or absence of moxifloxacin at the MIC.

The clinical antimicrobial moxifloxacin was employed to control bacterial load and extend the HCE-T scratch/infection assay, allowing observation of potential compound effects after 24 h. Without antibiotic present, the bacteria overwhelmed the cell culture and were cytotoxic to HCE-T cells after 24 h (Supplementary Figure S6). Moxifloxacin, at an minimal inhibitory concentration (MIC) of 2 µM (Figure 6A), limited the number of colony forming units (CFU) of bacteria in the culture medium after 24 h, whilst still enabling T3SS and ExoU mediated cytotoxicity to be detected (Figure 6B). Importantly, none of the potential ExoU inhibitors were bactericidal over 24 h and MICs could not be established, indicating that these inhibitors did not affect bacterial growth.

Live/Dead fluorescence microscopy was employed to visualise the scratched HCE-T cells 24 h after infection in the presence of moxifloxacin, at the MIC, and varying concentrations of ExoU inhibitors (Figure 6D). In the presence of moxifloxacin only (DMSO control), there was no apparent wound closure and the majority of the cells within the scratch margin had absorbed the ethidium homodimer dye (red cells), indicating extensive cell death, as a result of ExoU mediated cell lysis. In the presence of PSA and compound A with moxifloxacin (Figure 6D), cell lysis was limited to cells along the border of the scratch. The wound size also appeared to be reduced, and effect which was more prominent as compound concentrations were increased to 10 µM. Similar to PSA and compound A exposure, when scratched and infected HCE-T cells were treated with 0.5 µM compound B and 2 µM moxifloxacin for 24 h, cell lysis was reduced (compared with DMSO) and was only apparent along the scratch border. When the concentration of compound B was increased to 5 and 10 µM, we observed partial wound closure, with cells from either side of the scratch making contact (Figure 6D).

To quantify these data, the scratch surface area for each condition was measured (Figure 6E) and the percentage of viable cells within the scratch margin was calculated (Figure 6F). For scratched and infected cells exposed to 1 µM PSA, compound A or compound B (with moxifloxacin at the MIC), the total scratch surface area (mm^2^) was not significantly different from DMSO and moxifloxacin exposed cells. At a concentration of 5 µM, only compound B afforded a significant reduction in total scratch surface area compared with DMSO (72% ± 19%, *P* = 0.006). However, at compound concentrations of 10 µM, both compound A and compound B caused reductions in total scratch surface area (35% ± 12% and 83% ± 17%). PSA, compound A and compound B, with moxifloxacin, were all effective in reducing cell death, induced by PA103 ΔUT: ExoU infection. Greater percentages of viable HCE-T cells were detected within the scratch margin when these compounds were present, compared with DMSO plus moxifloxacin (Figure 5F).

The measurement of LDH release in HCE-T cells 24 h post infection of scratched HCE-T cells with PA103 ΔUT: ExoU, complemented the fluorescence microscopy (Figure 5G). Compound dose response experiments demonstrated that PSA, compound A and compound B used in combination with 2 µM moxifloxacin were far more effective at protecting HCE-T cells from lysis than these compounds or moxifloxacin alone (Figure 5G). Combination of compounds A and B with moxifloxacin led to the greatest reduction in LDH release (Figure 5G), 67% and 76%, respectively at a concentration of 10 µM.

### Molecular docking simulations of compounds to ExoU

**Figure 7.**
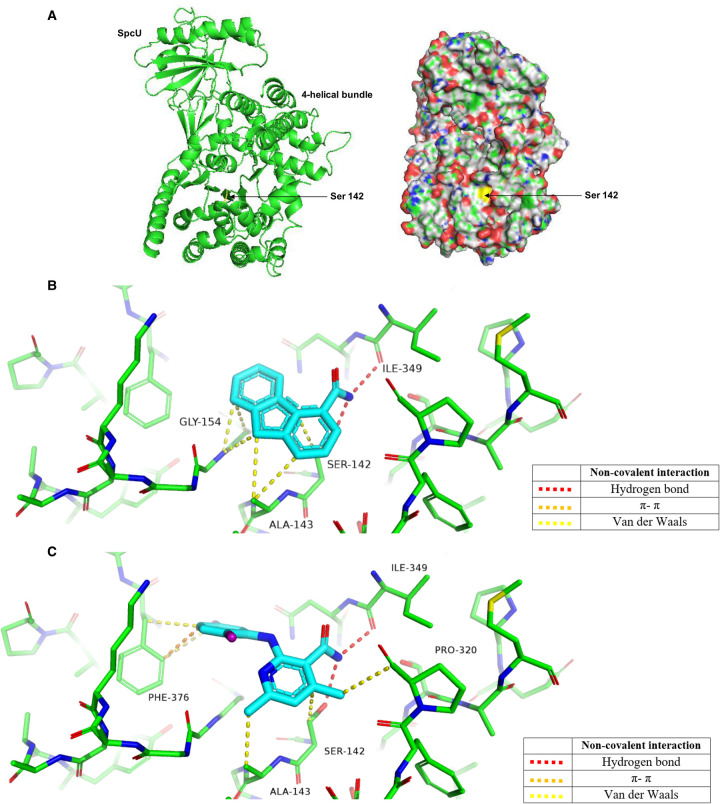
Docking poses of PSA and compound B to ExoU. (**A**) Structure and Connolly surface of the ExoU-SpcU complex with the catalytic serine 142 residue highlighted in yellow. Docked molecules (**B**) PSA and (**C**) compound B are rendered as sticks (carbon — cyan, nitrogen — blue, oxygen — red, chlorine — purple). Residues involved in non-covalent interactions are rendered as thin sticks (carbon — green, nitrogen — blue, oxygen — red). Non-covalent contacts are shown as dotted lines with the colour code given in the key. Non-covalent contacts analysed with ViewContacts software. Figure rendered in PyMol.

PSA, compound A and compound B displayed characteristics of competitive inhibition of ExoU *in vitro* phospholipase activity (Figure 1B). Using the crystal structure of ExoU, co-crystallised with its cognate chaperone SpcU (PDB: 3TU3) [19], we performed molecular docking simulations to visualise potential compound-ExoU interactions (Figure 7). The Connolly surface of ExoU revealed a potential substrate binding the pocket, adjacent to the catalytic Serine 142 residue (Figure 7A, yellow), as a potential ligand docking site. We observed that certain compounds (PSA in Figure 7B and compound B in Figure 7C) could dock with favourable energetics into this solvent-exposed region. All the highest-scoring docking solutions revealed that the compounds tested had similar poses.

Both PSA and compound B are predicted to be bound by a large number of cooperative non-covalent interactions (Figure 7). In both cases, the amide group makes a hydrogen bond interaction with Ile349 (red). Compound B has aromatic π−π stacking interactions with Phe376 (orange) and it also shows multiple van der Waals interactions with Phe376, Ala143, Ser142 and Pro320. PSA also displays multiple van der Waals interactions with Gly154, Ser142 and Ala143 with a hydrogen bond-π interaction between the amide of Gly154 and the aromatic ring of PSA.

## Discussion

In the present work, a pipeline from enzyme assay to the clinically relevant HCE-T scratch infection cell model has been assembled. The first major challenge was the poor yield of recombinant ExoU, and the co-induction of two contaminants of similar molecular mass. The second was the infection scratch assay itself and the use of a clinically relevant antibiotic, moxifloxacin to control bacterial growth and prevent the assay being swamped by bacteria depleting the cell culture medium. Put together, the pipeline has enabled new insights into small molecules previously described as inhibitors of ExoU and provides a means to undertake future screens of compound libraries to identify potential drug leads. Moreover, our methods of analysis can readily be directed to other tissues affected by infections of various *P. aeruginosa* strains that produce ExoU.

### Biochemical *in vitro* analysis of ExoU with prospective small molecule inhibitors

The poor yield of recombinant ExoU fusion proteins may be due to the toxicity of ExoU and it appears that a consequence is the co-expression of two endogenous proteins which on IMAC co-purify with ExoU (Figure 1A). An important contributing factor to the poor yield is its degradation by bacterial proteases shortly after induction of expression. Thus, previous purification procedures have adopted a short 3 h induction of ExoU expression followed by immediate purification from BL21(DE3)pLysS *E. coli* [24,26,32,33]. Alternate tagged variants, including glutathione-S-transferase (GST) and maltose-binding protein (MBP) ExoU fusion proteins, were also quickly degraded, which was apparent from a large abundance of GST/MBP proteins with ExoU cleaved away after respective pull-downs during purification (data not shown). In this study, we employed the serine protease inhibitor PMSF, which was added to C43 *E. coli* upon induction of His-tagged ExoU expression. This allowed ∼5-fold greater yields of recombinant His-ExoU. The two similar molecular mass contaminating proteins seen during purification of ExoU, were identified by mass spectrometry. These were bifunctional polymyxin resistance protein ArnA and glutamine-fructose-6-phosphate aminotransferase, which have previously been documented as contaminants in IMAC purifications from *E. coli* [41] (Figure 1A). The *arnA* gene encodes a 74.3 kDa bifunctional enzyme (UDP-l-Ara4N formyltransferase/UDP-GlcA C-4″-decarboxylase), which is involved in the modification of the lipid A with 4-amino-4-deoxy-l-arabinose to confer to resistance to cationic antimicrobial peptides and antibiotics, including polymyxin [42]. ExoU localises to cellular membranes via its 4 helical bundle domain and is fully activated by eukaryotic cofactors, but mechanisms and conformational rearrangements relevant to membrane binding and catalytic activity are not yet fully understood. ExoU is toxic to *E. coli* if it is co-expressed with ubiquitin [25,43]. Our data suggest that bifunctional polymyxin resistance protein ArnA and glutamine-fructose-6-phosphate aminotransferase could be induced in response to ExoU expression, perhaps to mitigate cell wall stress while ExoU is degraded by bacterial proteases.

### Exou inhibitors for biochemical and mechanistic analysis

The Cayman Chemical cPLA2 Assay Kit has previously been employed to detect the phospholipase activity of recombinant ExoU [27,28,39]. For our analysis of ExoU phospholipase activity and to test a small panel of previously proposed ExoU inhibitors along with certain clinical human phospholipase inhibitors, we adapted this protocol by sourcing individual reagents and making the assay compatible with 96- and 384-well plate formats. This allowed us to decrease reagent use, including ExoU, and increase the throughput of the assay so that it may be applicable to screening large compound libraries, which we believe should be a future focus to potentially discover novel small molecule inhibitors of ExoU. The previously identified ExoU inhibitors PSA, compound A and compound B (Figure 1C) exhibited low micromolar IC_50_ values for inhibition similar to those previously observed [39]. The arylsulfonamide compound, previously discovered from an independent cellular-based screen [29], did not inhibit ExoU phospholipase activity *in vitro*. This does not rule out the possibility that this compound prevents ExoU mediated toxicity, by *P. aeruginosa*, through mechanisms independent of ExoU catalytic activity inhibition, at least in yeast cells. As well as screening, future biochemical experiments should aim to explore the mechanisms of ExoU inhibition *in vitro* and in cells. Certain compounds may have the potential to target specific ExoU conformations or prevent interaction with activating cofactors [24,28]. To this end, structural biological studies could be performed to aid structure-guided design to improve the inhibitory activities and potency of compounds such as PSA, compound A and compound B. *In silico* molecular docking simulations of prospective ExoU small molecule inhibitors to the currently solved crystal structures of the SpcU-ExoU complex, 4AKX [20] and 3TU3 [19], might give insight as to how these compounds may be optimised. Indeed, our molecular docking simulations suggest that current ExoU inhibitors (PSA, compound A and compound B) (Figure 6), bind to a solvent-exposed pocket in the catalytic domain, forming various polar interactions. However, it is not known to what extent the structure of ExoU in complex with its endogenous inhibitor SpcU reflects that of the active ExoU in the host cell. Therefore, co-crystal structures of ExoU with one of these compounds would be important in thue development of a robust structure–function analysis.

### Inhibition of ExoU expressed in transfected HeLa cells

Transfected mammalian cells undergo rapid cellular lysis when induced to express WT, but not S142A ExoU [31]. In the HeLa transfection model, PSA, compound A and compound B, but not arylsulfonamide 1, were able to mitigate cytotoxicity induced after ExoU expression. The effects observed with PSA, compound A and compound B were dose responsive and statistically similar. The data indicate that ExoU could be degraded (or at least partially) by cellular proteases, as ExoU stability was increased in the presence of PMSF but not MG132 (Supplementary Figure S4). It was previously observed that ExoU becomes ubiquitinated in mammalian cells at Lys178 [20,40]. This modification does not seem to influence the toxicity exerted by WT ExoU phospholipase activity [20]. Ubiquitinated S142A ExoU was found to be targeted to acidic organelles [20]. The inositol polyphosphate phosphatase SopB is a *Salmonella* type III effector, which becomes ubiquitinated at Lys6 after delivery to mammalian cells [44]. This modification also does not affect SopB stability or membrane association, but was found to extend temporal association with *Salmonella*-containing vacuoles (SCVs) [44]. It is yet to be fully explored whether or not ubiquitination of ExoU contributes to activation or molecular rearrangement, but there is evidence to suggest that ubiquitination of ExoU promotes endosomal association [20], which perhaps serves as a defence mechanism against ExoU mediated cytotoxicity.

S142A ExoU could be detected more readily in cellular lysates [40], perhaps due to the fact that WT ExoU expression was acutely lethal and thus could not accumulate to a level that was reasonably detectable in transfected HeLa cells (Figure 2A). Therefore, by inducing expression of S142A ExoU, in the presence of ExoU inhibitors, we observed that compounds A and B, but not PSA or arylsulfonamide 1, caused a reduction in the total amount of S142A ExoU (Figure 3). PSA, compound A and compound B possessed similar IC_50_ values for ExoU inhibition *in vitro* (Figure 1B) and had similar protective effects in WT ExoU expressing HeLa cells (Figure 2B–E). All three compounds possess the ability inhibit ExoU in HeLa cells, but compounds A and B may also promote degradation of ExoU by endogenous proteases.

### A scratch and infection assay to evaluate the therapeutic potential of ExoU inhibitors

Corneal cells of the human eye form an apical barrier, which is important for function and protection against infection [45]. Infections usually arise after physical damage to the cornea from opportunistic pathogens [45,46]. We found that fully confluent HCE-T cells were not susceptible to infection by PA103 (Figure 4A), but sub-confluent cells (∼90%) were. By applying a scratch to the bottom of the well of fully confluent monolayer of HCE-T cells, we could simulate injury and allow T3SS and ExoU mediated cytotoxicity from PA103, which propagated over time from the periphery of the scratch, extending outwards. This movement of infection, spreading from the border of the scratch and towards neighbouring cells, may offer insights into the mechanisms of infection and disease progression *in vivo*. Not only could we simulate injury in this way, the scratch had the advantage of providing a point of focus from which to observe the effects of ExoU mediated cytotoxicity and potential protective effects of antibiotics and prospective ExoU small molecule inhibitors. This was essential for quantification of the effects of inhibitors. In this scratch and infection assay, we were able to visualise tissue damage exerted by the PA103 T3SS, quantify the wound surface area and calculate the percentage of viable cells after various treatments. In the scratch and infection assay, PSA, compound A and compound B were effective in the low micromolar region, with significant protection of HCE-T cells from PA103 infection by compound B at 0.5 µM (Figure 5). The toxicity analysis in HCE-T cells indicated that all these compounds were well tolerated in the high micromolar concentrations (>30 µM) (Figure 4C), suggesting that with topical administration, ExoU inhibitors might achieve effective inhibitory concentrations.

The scratch assay was extended by employing moxifloxacin, a fluoroquinolone commonly used to treat eye infections that inhibits bacterial DNA synthesis [8], at an established MIC to manage bacterial load, whilst still maintaining the effects of ExoU mediated cytotoxicity (Figure 6B). We believe that this assay could be employed as a useful tool to analyse the effects of T3SS cytotoxicity in cell culture models for longer times. Using moxifloxacin to manage bacterial load in scratch and infection assays may also offer insights into mechanisms of disease progression and how infections could respond to extended treatments. Only when used in combination with moxifloxacin were PSA, compounds A and compound B effective at protecting HCE-T cells from infection over 24 h (Figure 6). This is likely due to the bacteria, when incubated with HCE-T cells for 24 h, growing exponentially leading to cytotoxic effects independent of ExoU expression [47]. In the instance of combinational treatment of compound B (10 µM) and moxifloacin (at the MIC), partial wound closure was observed (Figure 6D), whereas 10 µM compound A and to a lesser extent with 10 µM of PSA with moxifloxacin, a narrowing, but not closure, of the scratch was observed. The data, therefore, suggest that compounds A and B could be more effective than PSA at protecting HCE-T cells from ExoU for longer treatment courses. The stability of S142A ExoU was only significantly decreased by compounds A and B, but not PSA, in HeLa cells (Figure 3), despite the three compounds possessing similar IC_50_ values for *in vitro* inhibition of ExoU phospholipase activity (Figure 1C).

The present work provides a pipeline for the identification and analysis of ExoU inhibitors in a range of assays spanning *in vitro* enzyme activity to the protection of mammalian cells by ExoU mediated cytotoxicity using small molecule inhibitors. The data acquired using this pipeline also suggest that pharmacological targeting of ExoU may be compatible with antibiotic usage, whereby inhibitors of ExoU serve as an adjuvant therapy. Thus, the last stage of our pipeline will moreover allow *ex vivo* screens of ExoU inhibitors alone and as an adjuvant in combination with antibiotics, prior to embarking on low throughput *in vivo* screens necessary for future clinical translation. As these ExoU inhibitors were not bactericidal, we envision that, in a therapeutic context, ExoU inhibitors would serve to mitigate the rapid toxicity induced by ExoU, while antibiotics clear the infection.

## Data Availability

The datasets generated during and analysed during the current study are available from the corresponding author on reasonable request.

## Open Access

Open access for this article was enabled by the participation of University of Liverpool in an all-inclusive *Read & Publish* pilot with Portland Press and the Biochemical Society under a transformative agreement with JISC.

## Abbreviations

ATPC: arachidonoyl thio-phosphatidylcholine
CFU: colony forming units
DMSO: dimethylsulfoxide
GST: glutathione-S-transferase
ICUAP: intensive care unit-acquired pneumonia
IMAC: immobilised metal affinity chromatography
IPTG: isopropyl-β-d-thiogalactopyranoside
LDH: lactate dehydrogenase
MBP: maltose-binding protein
MIC: minimal inhibitory concentration
OP: oleyoxylethyl phosphorylcholine
PBS: phosphate-buffered saline
PIP_2_: phosphatidylinositol 4 5-bisphosphate
QD: quinacrine dihydrochloride
SEC: size-exclusion chromatography
T3SS: type 3 secretion system
