# Supplementary material for: A pipeline to evaluate inhibitors of the Pseudomonas aeruginosa exotoxin U
Source: Biochem J. 2021 Feb 12;478(3):647–68. doi: 10.1042/BCJ20200780 (PMC7886320; doi:10.1042/BCJ20200780)
Supplement: Supplementary Figures S1-S6 [file BCJ-478-647-s1.pdf]

Supplementary figures

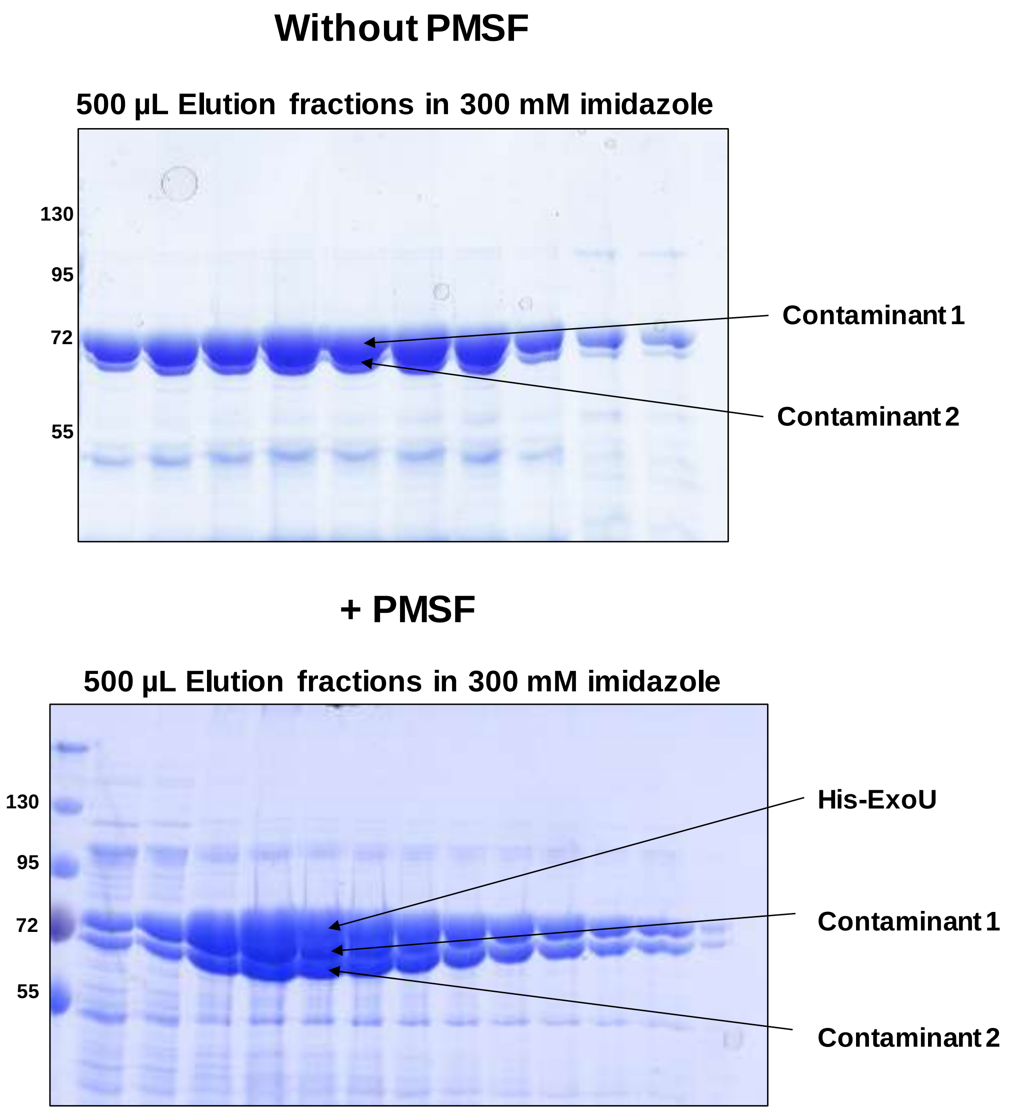

Supplementary Figure 1: Expression of His-tagged ExoU in the presence of PMSF. C43(DE3) E. coli were grown to 0.8 OD600 followed by the addition of IPTG and 100 µM PMSF. ExoU was expressed for 3 hours with the addition of 100 µM PMSF at each hour before centrifugation of E. coli and purification by immobilised metal affinity chromatography (IMAC).

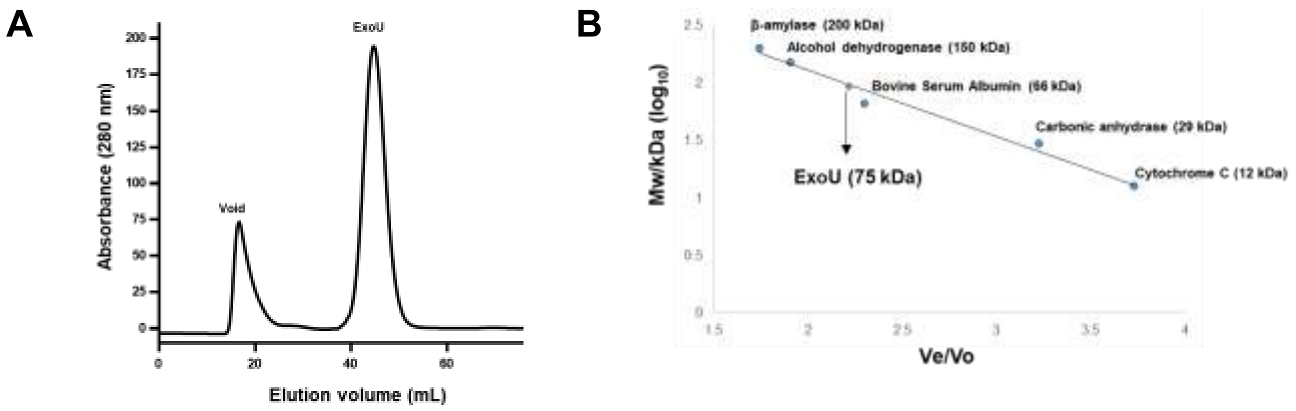

Supplementary figure 2: Size exclusion chromatography analysis of ExoU. (A) Purified His-tagged ExoU analysed by size exclusion chromatography. 2 mg of ExoU was resolved on a S200/60 gel filtration column with protein elution apparent from UV absorbance, detected at 280 nm. (B) Molecular weight standards dextran blue, β-amylase, alcohol dehydrogenase, albumin from bovine serum, carbonic anhydrase and cytochrome C were resolved on a S200/60 gel filtration column in order to obtain a standard comparison for purified ExoU analysis.

A

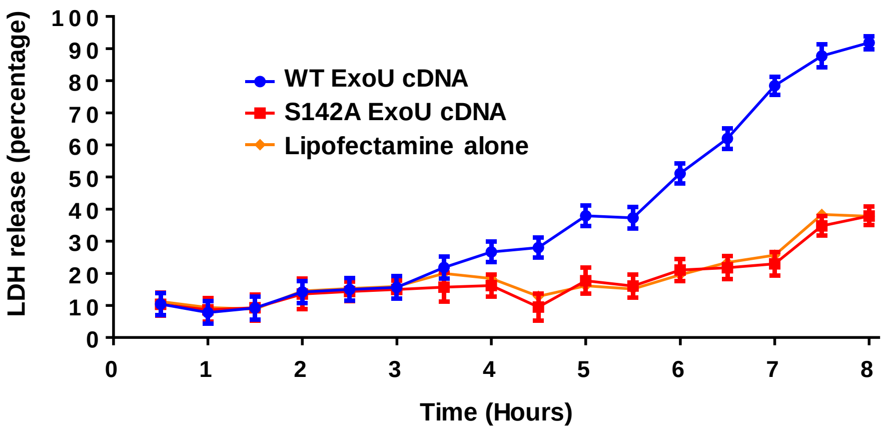

B

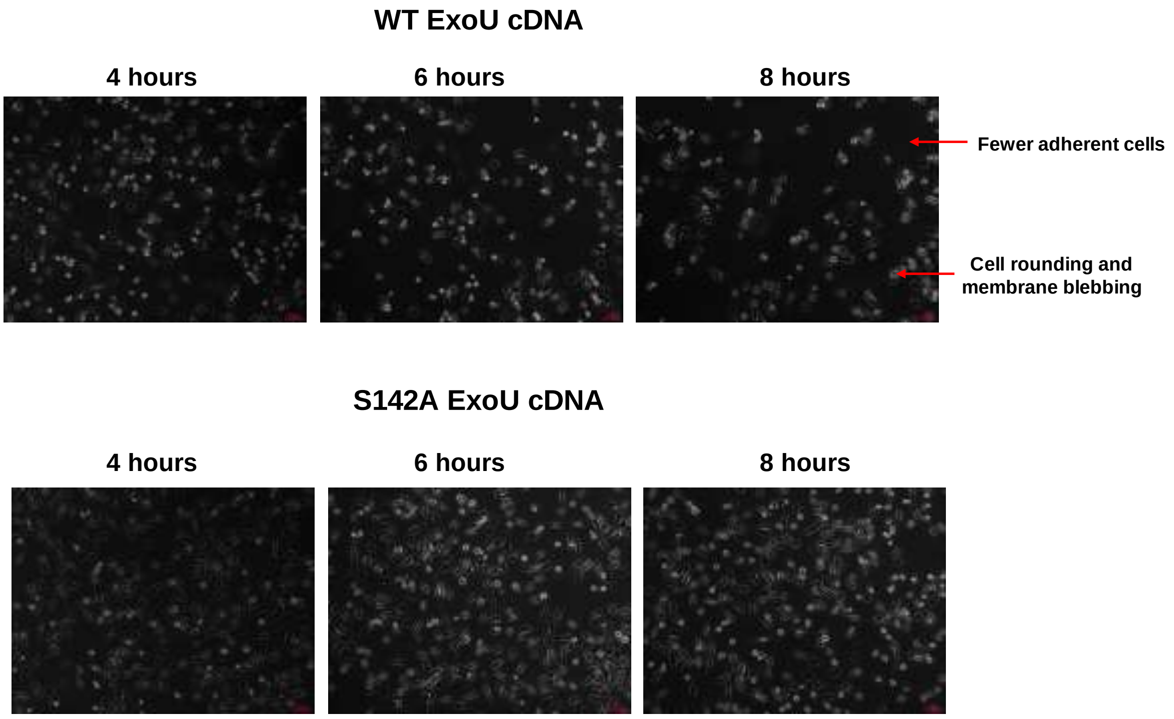

Supplementary Figure 3: Effect of WT and S142A ExoU expression on HeLa cell viability. HeLa cells were seeded in wells of a 6-well plate for 24 hours. Lipofectamine was used to transfect pcDNA5/FRT/TO encoding WT or S142A ExoU cDNA (1 µg per well) for 12 hours, followed by the addition of 10 µg/ml of tetracycline to induction of ExoU expression. (A) LDH assay time course analysis of HeLa cells transfected to express WT ExoU (blue) or S142A ExoU (red). (B) Brightfield images taken of transfected HeLa cells after induced expression of either WT or S142A ExoU at the indicated time points.

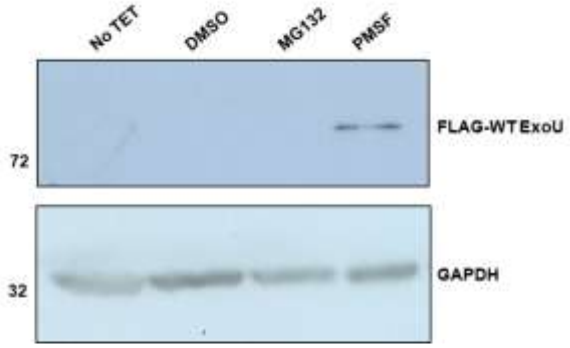

Supplementary Figure 4: Detection of FLAG-tagged WT ExoU in transfected HeLa cells. HeLa cells were seeded in wells of a 6-well plate for 24 hours. Lipofectamine was used to transfect 1 µg of pcDNA5/FRT/TO encoding WT ExoU cDNA for 12 hours, followed by the addition of 10 µg/ml of tetracycline to induce FLAG-WT ExoU expression. Western blot analysis reveals the total abundance of FLAG-tagged WT-ExoU in the presence of DMSO, 50 µM MG132 or 50 µM PMSF after 4 hours incubation.

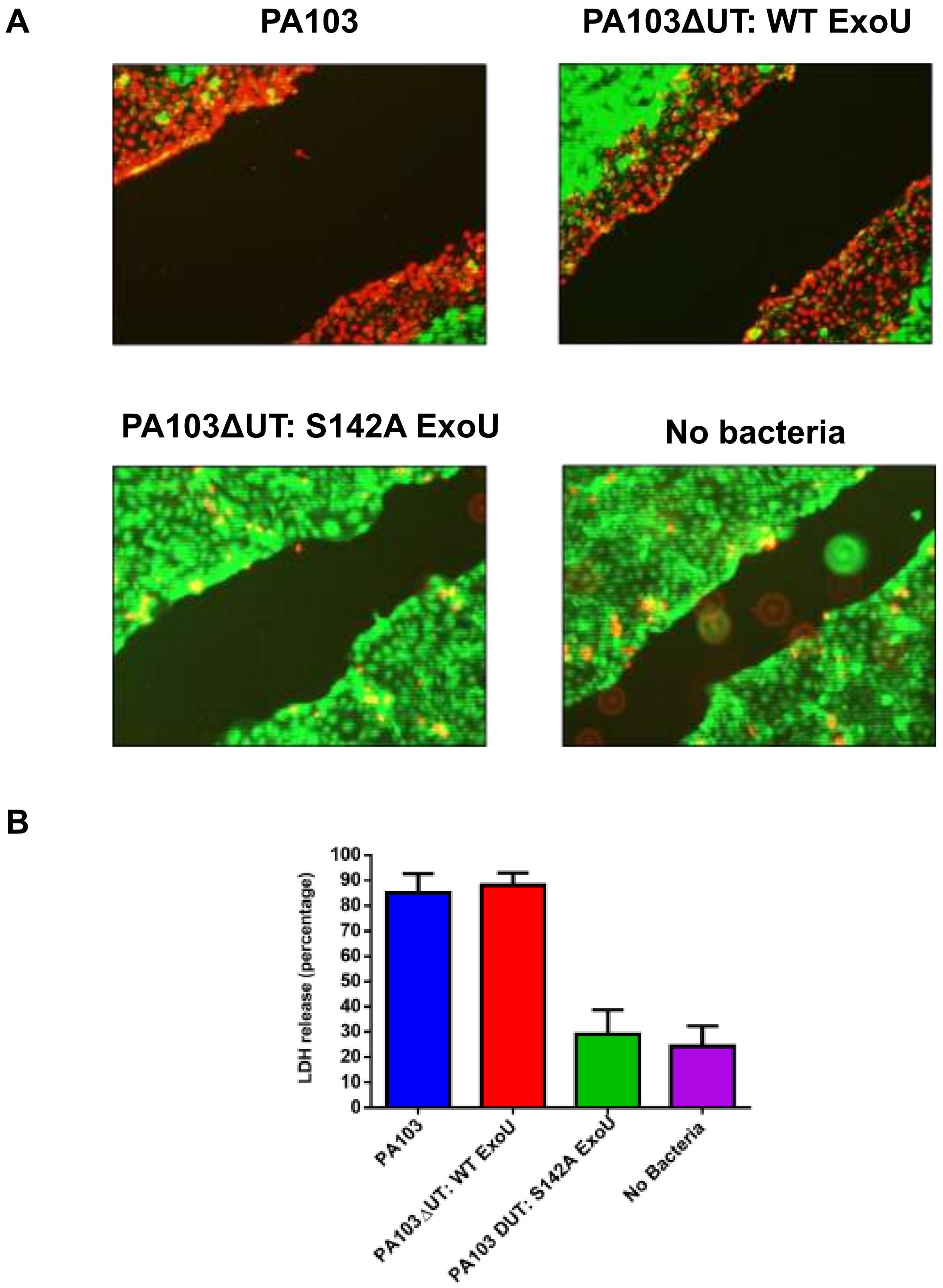

Supplementary Figure 5: Infection of scratched HCE-T cells with PA103 mutants. HCE-T were gown to full confluence and a scratch applied to the well bottom prior to infection with either PA103, PA103 ΔUT: ExoU or PA103 ΔUT: S142A ExoU at an MOI of 2.5 for 6 hours, followed by analysis by Live/Dead fluorescence microscopy (A) or LDH release (B).

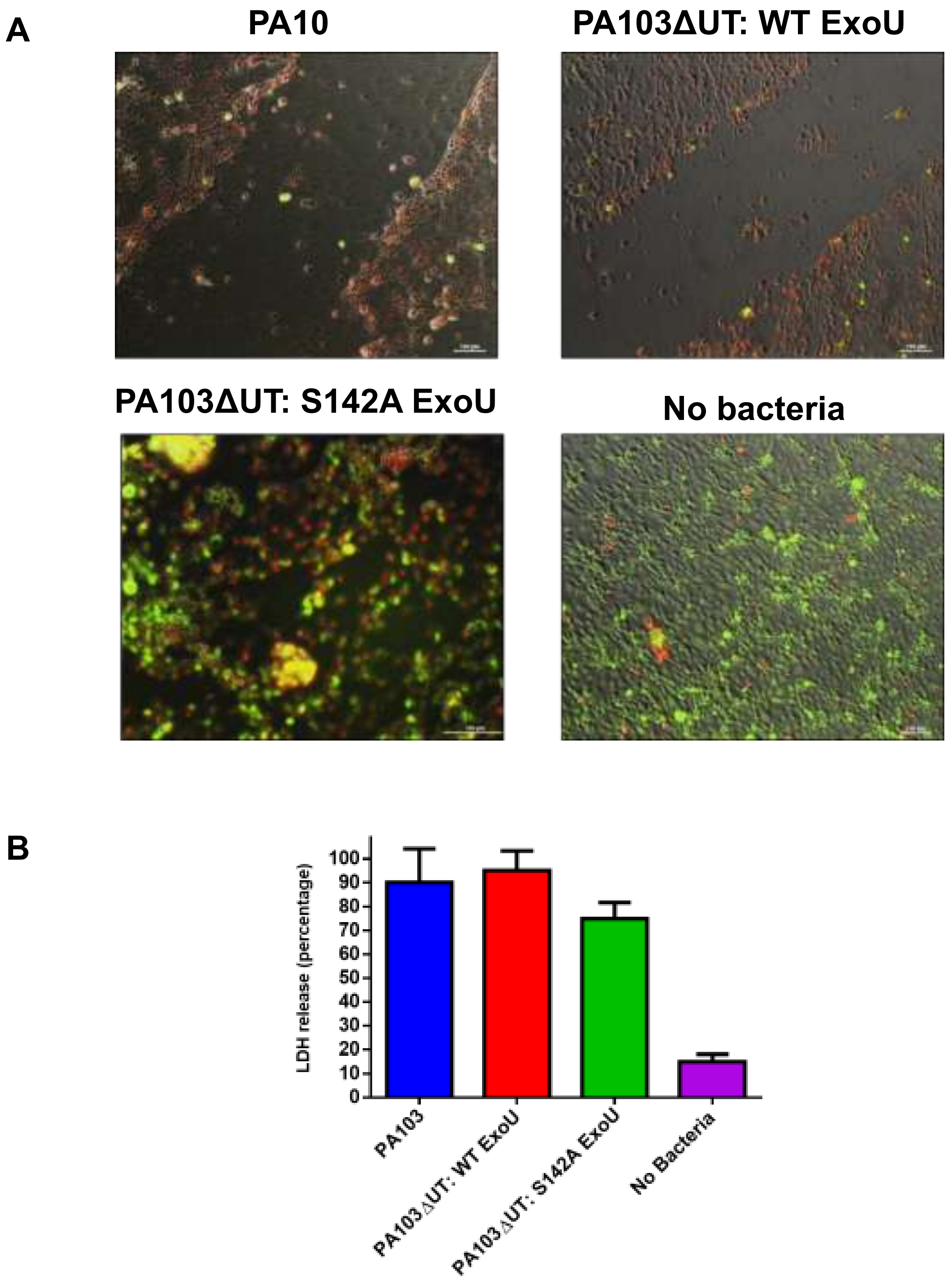

Supplementary Figure 6: Toxicity of PA103 mutants to scratched HCE-T cells after 24 hours incubation without antibiotic present. HCE-T were gown to full confluence and a scratch applied to the well bottom prior to infection with either PA103, PA103 ΔUT: ExoU or PA103 ΔUT: S142A ExoU at an MOI of 2.5 for 24 hours, followed by analysis by Live/Dead fluorescence microscopy (A) or LDH release (B).
